# Prevalence and Risk Factors of Venous Thromboembolism in Critically Ill Patients with Severe COVID-19 and the Association between the Dose of Anticoagulants and Outcomes

**DOI:** 10.2478/jccm-2022-0023

**Published:** 2022-11-12

**Authors:** Hasan M. Al-Dorzi, Muhannad Q. Alqirnas, Mohamed M. Hegazy, Abdullah S. Alghamdi, Mohammed T. Alotaibi, Mohammed T. Albogami, Mohammed M. Alhafi, Salem Alwadani, Ashraf Elsharkawi, Yaseen M. Arabi

**Affiliations:** 1 College of Medicine, King Saud bin Abdulaziz University for Health Sciences, King Abdullah International Medical Research Center, and Intensive Care Department, King Abdulaziz Medical City, Ministry of National Guard Health Affairs, Riyadh, Saudi Arabia

**Keywords:** COVID-19, anticoagulation, heparin, thromboprophylaxis

## Abstract

**Introduction:**

COVID-19 is characterized by a procoagulant state that increases the risk of venous and arterial thrombosis. The dose of anticoagulants in patients with severe COVID-19 pneumonia without suspected or confirmed thrombosis has been debated.

**Aim of the study:**

We evaluated the prevalence, predictors, and outcomes of venous thromboembolism (VTE) in critically ill COVID-19 patients and assessed the association between the dose of anticoagulants and outcomes.

**Materials and methods:**

This retrospective cohort included patients with COVID-19 who were admitted to the ICU between March and July 2020. Patients with clinically suspected and confirmed VTE were compared to those not diagnosed to have VTE.

**Results:**

The study enrolled 310 consecutive patients with severe COVID-19 pneumonia: age 60.0±15.1 years, 67.1% required mechanical ventilation and 44.7% vasopressors. Most (97.1%) patients received anticoagulants during ICU stay: prophylactic unfractionated heparin (N=106), standard-dose enoxaparin (N=104) and intermediate-dose enoxaparin (N=57). Limb Doppler ultrasound was performed for 49 (15.8%) patients and chest computed tomographic angiography for 62 (20%). VTE was diagnosed in 41 (13.2%) patients; 20 patients had deep vein thrombosis and 23 had acute pulmonary embolism. Patients with VTE had significantly higher D-dimer on ICU admission. On multivariable Cox regression analysis, intermediate-dose enoxaparin versus standard-dose unfractionated heparin or enoxaparin was associated with lower VTE risk (hazard ratio, 0.06; 95% confidence interval, 0.01-0.74) and lower risk of the composite outcome of VTE or hospital mortality (hazard ratio, 0.42; 95% confidence interval, 0.23-0.78; p=0.006). Major bleeding was not different between the intermediate- and prophylactic-dose heparin groups.

**Conclusions:**

In our study, clinically suspected and confirmed VTE was diagnosed in 13.2% of critically ill patients with COVID-19. Intermediate-dose enoxaparin versus standard-dose unfractionated heparin or enoxaparin was associated with decreased risk of VTE or hospital mortality.

## Introduction

Severe Coronavirus Disease 2019 (COVID-19) is characterized by hyperinflammation accompanied by a procoagulant state [1, 2, 3]. In addition to this state, critically ill patients with COVID-19 frequently have other factors, such as antecedent comorbidities, immobility, sedation, central venous lines and mechanical ventilation, that increase the risk of venous thromboembolism (VTE) and other thrombotic events. The studies that evaluated the epidemiology of VTE in COVID-19 patients have showed variable results, likely due to differences in methodology and case mix [4, 5, 6, 7, 8, 9]. A systematic review and meta-analysis in which 15 standard sources and COVID-19-specific sources were searched between January 1, 2020, and July 31, 2020 found that the pooled incidence was 17.0% (95% confidence interval [CI], 13.4-20.9%) for VTE, 12.1% (95% CI, 8.4-16.4%) for deep vein thrombosis (DVT) and 7.1% (95% CI, 5.3-9.1%) for pulmonary embolism (PE) [10]. The VTE incidence was higher among patients in the intensive care unit (ICU) (27.9% versus 7.1% in the ward) [10]. Whether COVID-19 has higher VTE rates than other severe infections is nevertheless unclear. In critically ill patients with sepsis, VTE incidence was as high as 37% in one prospective study where 80.5% of patients received pharmacologic prophylaxis and 19.5% sequential compression devices because of a contraindication for anticoagulants [11]. Predictors of thrombosis in COVID-19 patients include age, prolonged prothrombin time and partial thromboplastin time (PTT), higher D-dimer, and central venous lines [5, 7, 9].

Anticoagulation at different intensities (standard prophylactic, intermediate or therapeutic doses) have been advocated for patients with COVID-19. Higher intensity anticoagulation was suggested in patients with increased D-dimers and worsening hepatic, renal or respiratory function [6, 12, 13, 14] This suggestion was based on the high prevalence of hypercoagulability and high rates of VTE in patients with COVID- 19 with early observational studies showing benefits associated with the use of anticoagulants [15]. A retrospective study from China found lower 28‐day mortality with heparin use (mostly prophylactic enoxaparin) than non-use in patients with sepsis-induced coagulopathy score ≥ 4 (40.0% versus 64.2%, p=0.03), and when D‐dimer was > 6 fold the upper limit of normal (32.8% versus 52.4%, p=0.017) [15]. Another study found lower 30-day mortality among the 3627 patients who received prophylactic anticoagulation (14.3%; 95% CI, 13.115.5%) compared with the 697 patients who did not (18.7%; 95% CI, 15.1-22.9%) [16]. Higher enoxaparin doses (0.62±0.16 mg/kg) has been associated with a better thromboprophylactic action (hazard ratio, 0.2; p=0.04) compared with lower doses [17]. In another retrospective study, a multivariable regression analysis showed that intermediate- compared to prophylactic-dose anticoagulation was associated with a significantly lower cumulative incidence of in-hospital death (hazard ratio, 0.518; 95% CI, 0.308-0.872) among propensity score-matched patients (N=382) [18]. However, a large multicenter observational cohort study of 2809 critically ill patients with COVID-19 in the US found no benefit of anticoagulation at therapeutic doses initiated within 2 days of ICU admission compared with standard-dose thromboprophylaxis [19]. Multiple randomized trials have addressed different anticoagulants at different doses in COVID-19. So far, the published ones have not demonstrated significant benefits from anticoagulation at intermediate or therapeutic doses compared with standard doses in critical COVID-19 [20, 21, 22].

The objectives of this study were to determine the prevalence of clinically suspected and confirmed VTE in critically ill patients with COVID-19, describe the thromboprophylaxis and anticoagulation practices and outcomes, and identify predictors of symptomatic VTE and mortality.

## Materials and Methods

### Design, patients and setting

This was a retrospective cohort study of adult (age ≥ 18 years) patients with critical COVID-19 who were admitted to the noncardiac ICUs of King Abdulaziz Medical City in Riyadh between March 1 and July 31, 2020 (consecutive nonprobability sample). The study period covered the first wave of COVID-19 in Riyadh, Saudi Arabia. A confirmed COVID-19 case was defined as a clinical presentation consistent with COVID-19 and detection of SARS-CoV-2 RNA in a respiratory specimen using a molecular amplification detection test such as rt-PCR [23]. Exclusion criteria included stay in the ICU < 24 hours. Critical COVID-19 was defined as having acute respiratory failure, septic shock and/ or multiple organ dysfunction [24].

In this study, the decision to use thromboprophylaxis (standard-dose unfractionated heparin (UFH) or enoxaparin or intermediate-dose enoxaparin) or therapeutic anticoagulation was at the discretion of the treating team. In our ICUs, enoxaparin was preferred over UFH for thromboprophylaxis unless patients had severe renal insufficiency (creatinine clearance < 30 ml/ min) [25]. Intermediate-dose enoxaparin was defined as 40 mg subcutaneously twice daily [26]. Weight-based higher doses may be given for patients with morbid obesity [26]. Therapeutic anticoagulation was defined as providing subcutaneous enoxaparin at 1 mg per kg twice daily or protocolized intravenous heparin infusion targeting activated PTT of 45-60 seconds if the patient had increased risk for bleeding and 60-85 seconds otherwise. During the study period, routine surveillance for DVT using Doppler ultrasound was not performed.

### Data

We collected data on patients’ demographics, premorbid conditions, severity of illness on ICU admission, admission laboratory findings, ICU interventions, including vasopressor therapy and invasive mechanical ventilation, and anticoagulant use and dose.

The primary outcome was the occurrence of clinically suspected and confirmed VTE, defined as lower limb DVT, PE, or both, which were suspected by the treating team and diagnosed by Doppler ultrasound (for DVT) or CT pulmonary angiography (for PE) [27]. The secondary outcomes were the composite of clinically suspected and confirmed VTE or hospital mortality, the occurrence of arterial thrombosis (stroke, bowel is-chemia, limb ischemia) and major bleeding (defined as clinically overt bleeding accompanied by a decrease in hemoglobin level by ≥ 20 g/L or transfusion ≥ 2 units of packed red cells, or resulting in shock requiring vasopressors), hospital mortality, ICU mortality, duration of mechanical ventilation, need for tracheostomy, length of stay in the ICU and hospital. We did not assess incidents of myocardial infarction in this study.

### Statistical analysis

In this study, patients were categorized into two groups: patients who had clinically suspected and confirmed VTE and those who were not diagnosed to have VTE. The descriptive statistics was presented as frequency and percentage for the categorical variables and the mean with standard deviation or the median with the first and third quartiles for numerical variables. We compared the baseline and outcome variables between the two groups using the Fisher’s exact test or Chi-square test for categorical variables and Student’s t test or Mann-Whitney U test for continuous variables as appropriate.

As D-dimer was suggested to be a predictor of VTE in COVID-19, we performed receiver operating characteristic (ROC) curve analysis and calculated the Youden index [28] to determine the best cut-off of admission D-dimer that discriminated between patients with confirmed VTE from those who did not have VTE. The same analysis was also performed for the admission D-dimer/fibrinogen ratio, which has been suggested to have better diagnostic characteristics than D-dimer in VTE [29].

We hypothesized that higher intensity anticoagulation (intermediate-dose enoxaparin versus prophylactic-dose UFH or enoxaparin) was associated with lower risk of VTE and death. Hence, Cox regression analysis was performed to assess the risk factors for clinically suspected and confirmed VTE. In the model, the following variables were entered: age, admission Sequential Organ Failure Assessment (SOFA) score, intermediate-dose enoxaparin versus standard-dose UFH or enoxaparin and variables with p-value between groups < 0.2 (history of asthma, central line location, admission D-dimer and fibrinogen, vasopressor therapy, and invasive mechanical ventilation). Cox regression analysis was also performed to determine the predictors of the composite outcome of clinically suspected and confirmed VTE or hospital mortality. The variables entered in the model were those with p-value < 0.2 between patients who had the composite outcomes and those who did not (age, admission SOFA score, admission Glasgow Coma Scale, hypertension, diabetes, congestive heart failure, chronic obstructive pulmonary disease, chronic kidney disease, previous VTE, central venous catheter location, hemoglobin, platelet count, serum creatinine, lactate, D-dimer, PTT, international normalized ratio, vasopressor therapy, mechanical ventilation, and the heparin dose category [standard dose, intermediate dose, and therapeutic dose]). The results were presented as hazard ratio with 95% CI. A test was considered significant if the p-value was < 0.05. The *Statistical Product and Service Solution (*SPSS) software version 15 was used for all analyses [30].

## Results

### Characteristics of patients

During the study period, 310 consecutive patients with COVID-19 were eligible and were included. Their characteristics are described in **Table 1**. The mean age was 60.1±15.1 years with 75.1% being males and 67.1% requiring intubation and mechanical ventilation.

**Table 1:** Characteristics of patients

	All patients N=310	VTE N=41	No VTE N=269	P value
Age (years), mean±SD	60.1±15.1	58.8±15.7	60.3±15.1	0.56
Male gender, N (%)	233 (75.2)	30 (73.2)	203 (75.5)	0.75

Body mass index* (kg/m2), median (Q1, Q3)	28.9 (25.0, 34.1)	27.1 (24.4, 33.3)	29.1 (25.1, 34.2)	0.32
No obesity < 30 kg/m2, N (%)	169 (55.6)	25 (62.5)	144 (54.5)	
Obesity 30-39.9 kg/m2, N (%)	110 (36.2)	10 (25.0)	100 (37.9)	0.22
Obesity ≥ 40 kg/m2, N (%)	25 (8.2)	5 (12.5)	20 (7.6)	

Comorbid conditions, N (%)				
Hypertension	173 (56.0)	22 (53.7)	151 (56.3)	0.75
Diabetes	173 (56.0)	22 (53.7)	151 (56.3)	0.75
Congestive heart failure	22 (7.1)	1 (2.4)	21 (7.8)	0.21
COPD	10 (3.2)	0 (0)	10 (3.7)	0.21
Bronchial asthma	18 (5.8)	6 (14.6)	12 (4.5)	0.02
Chronic kidney disease	36 (11.7)	3 (7.3)	33 (12.3)	0.35
Hemodialysis	11 (3.6)	11 (3.6)	9 (3.4)	0.63
Previous VTE, N (%)	11 (3.5)	1 (2.4)	10 (3.7)	1.0
History of thrombophilia	9 (2.9)	1 (2.4)	8 (3.0)	1.0
Prior anticoagulation	20 (6.5)	2 (4.9)	18 (6.7)	1.0
GCS on admission, mean±SD	12.3±4.5	11.5±4.9	12.5±4.4	0.21
SOFA on admission, mean±SD	6.1±3.9	6.5±3.9	6.0±3.9	0.52
SOFA at day 7, mean±SD	6.8±4.3	7.2±3.6	6.7±4.4	0.45

Pertinent laboratory findings on admission				
Creatinine (μmol/L), median (Q1, Q3)	93.0 (73.0, 144.5)	103.0 (71.5, 139.5)	93.0 (73.0, 146.8)	0.97
WBC x 109/L, median (Q1, Q3)	9.82 (6.65, 13.80)	10.80 (7.37, 16.10)	9.81 (6.51, 13.75)	0.22
Neutrophils	7.94 (4.95, 11.55)	7.82 (5.45, 11.70)	7.95 (4.88, 11.54)	0.51
Lymphocytes	0.93 (0.63, 1.41)	0.89 (0.62, 1.32)	0.94 (0.64, 1.42)	0.54
Admission hemoglobin (g/L), mean±SD	129±24	132±25	128±24	0.37
Admission platelets x 109/L, mean±SD	279±120	291±137	278±117	0.51
PTT in seconds, median (Q1, Q3)	29.1 (26.5, 32.6)	28.5 (26.0, 32.8)	29.3 (26.5, 32.6)	0.61
INR, median (Q1, Q3)	1.10 (1.04, 1.18)	1.12 (1.06, 1.30)	1.10 (1.03, 1.18)	0.12
Lactate (mmol/L), mean±SD	2.6±2.4	3.1±3.2	2.5±2.3	0.31
Lactate dehydrogenase (U/L), median (Q1, Q3)	553.0 (430.0, 749.0)	615.0 (412.0, 837.0)	544.5 (437.5, 731.5)	0.23
Fibrinogen (g/L), median (Q1, Q3)	5.22 (3.81, 6.92)	4.05 (2.62, 6.07)	5.49 (3.97, 7.06)	0.003
D-Dimer (mg/L), median (Q1, Q3)	1.70 (0.80, 4.05)	3.86 (1.28, 14.43)	1.47 (0.77, 3.72)	0.001
D-Dimer/fibrinogen ratio	1.9±5.3	4.2±8.7	1.5±4.5	0.14

Key interventions in the ICU before VTE				
Central venous catheter	206 (66.5)	33 (80.5)	173 (64.3)	0.04
Internal jugular	156 (50.3)	26 (63.4)	130 (48.3)	
Subclavian	13 (4.2)	3 (7.3)	10 (3.7)	0.20
Femoral	34 (11.0)	4 (9.8)	30 (11.2)	
Vasopressor use, N (%)	138 (44.7)	27 (67.5)	111 (41.3)	0.002
Invasive mechanical ventilation, N (%)	208 (67.1)	33 (80.5)	175 (65.1)	0.05
PaO2/FiO2 ratio before intubation, median (Q1, Q3)	92.5 (66.5, 164.3)	90.0 (78.7, 214.5)	95.8 (63.0, 153.0)	0.17
Renal replacement therapy, N (%)	67 (21.6)	11 (26.8)	56 (20.8)	0.38

APACHE: Acute Physiology and Chronic Health Evaluation; COPD: chronic obstructive pulmonary disease; CT: computed tomography; GCS: Glasgow Coma Scale; INR: International Normalized Ratio; *PaO2/ FiO2 ratio* is the ratio of arterial oxygen partial pressure (PaO2 in mmHg) to fractional inspired oxygen; PTT: Partial Thromboplastin Time; Q1: first quartile; Q3: third quartile; SD: standard deviation; SOFA: Sequential Organ Failure Assessment; VTE: venous thromboembolism. Variables with skewed distribution were presented as median with the first third quartiles. The Mann-Whitney U test was used to compare the two groups. *6 patients had missing BMI.

### Use of anticoagulants

Most (97.1%) patients received anticoagulants during ICU stay. The different regimens are described in **Table 2**. Enoxaparin was more commonly used than UFH (59.7% versus 37.1%) and standard-dose anticoagulants (median daily dose= 40 mg, corresponding to 0.5 mg/kg) more than intermediate (median daily dose= 80 mg/ day, corresponding to 1 mg/kg) and therapeutic doses. Therapeutic anticoagulation was used in 33 patients (8 as continuation of prior anticoagulation and 25 as empirical therapy for suspected VTE).

**Table 2:** VTE prophylaxis practices

Variables	All patients N=310	VTE N=41	No VTE N=269	P value
No anticoagulant prophylaxis, N (%)	9 (2.9)	3 (7.3)	6 (2.2)	0.10
UFH, N (%)	115 (37.1)	15 (36.6)	100 (37.2)	0.94
Standard dose (5000 U 12 hrly)	53 (46.1)*	8 (53.3)*	45 (45.0)*	0.59
standard dose (5000 U 8 hrly)	53 (46.1)*	4 (26.7)*	49 (49.0)*	0.16
Intravenous infusion	9 (7.8)*	3 (20.0)*	6 (6.0)*	0.09
LMWH, N (%)	185 (59.7)	23 (56.1)	162 (60.2)	0.62
standard dose*	104 (56.2)*	11 (47.8)*	93 (57.4)*	0.50
Intermediate dose*¶	57 (30.8)*	3 (13.0)*	54 (33.3)*	0.055
Therapeutic dose*	24 (13.0)*	9 (39.1)*	15 (9.3)*	<0.001

*The denominator used for calculating the percentage is the total number of patients receiving either UFH or LMWH; ¶ Intermediate dose enoxaparin was mostly 40 mg every 12 hours; LMWH: low-molecular weight heparin, UFH: Unfractionated heparin, VTE: venous thromboembolism

### Thrombotic events

There were 41 VTE cases (13.2%; 95% CI, 9.7-17.5%) and 19 arterial thrombotic events (6.1%; 95% CI, 3.79.4%). VTE cases were diagnosed on a median of 17 days (Q1, Q3: 5, 25) after hospital admission. DVT was diagnosed in 20 patients out of 52 (38.5%) who had Doppler ultrasound of limbs (14 lower limb and 6 upper limb). PE was diagnosed in 23 patients out of 61 (37.7%) who had CT Chest angiography. The location was the main artery in four patients, lobar in one, segmental in 11 and subsegmental in 7. The 19 arterial thrombotic events consisted of 15 cases of acute stroke and 4 cases of acute limb ischemia. VTE was diagnosed in 6/19 (31.6%) patients with arterial thrombosis.

**Fig. 1:**
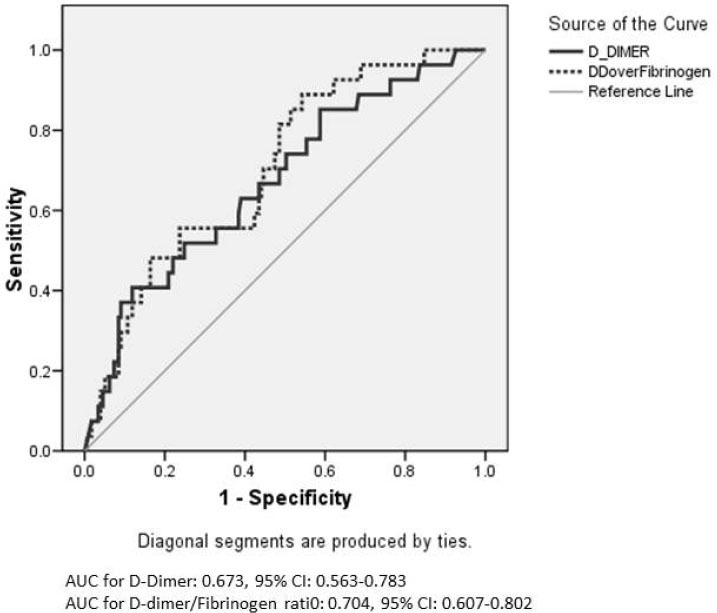
Receiver operating characteristic (ROC) curve for admission D-dimer and D-dimer/fibrinogen ratio for the diagnosis of clinically suspected and confirmed venous thromboembolism

The characteristics of patients who were diagnosed to have VTE and those who were not are shown in **Table 1**. VTE was more prevalent in patients who required mechanical ventilation and vasopressors. Patients with VTE had higher admission D-dimers and lower fibrinogen levels compared with those who were not diagnosed to have VTE. However, ROC curve analysis found that D-dimer and D-dimer/fibrinogen ratio had modest ability to differentiate patients with VTE from those without VTE (**Figure 1**). The best thresholds were 10.6 mg/ L and 0.26, respectively.

For patients receiving UFH at prophylactic doses (N=106), VTE was diagnosed in 15.1% of patients receiving 5000 U 12 hourly and 7.5% of those receiving 5000 U 8 hourly (p=0.22). For patients receiving prophylactic enoxaparin (N=161), VTE was diagnosed in 10.6% of patients on standard dose and 5.3% of patients on intermediate dose (p=0.38). The multivariable Cox regression analysis showed that vasopressor therapy was associated with increased VTE risk (hazard ratio, 6.69; 95% CI,1.26-35.52), while intermediate-dose enoxaparin versus standard-dose UFH or enoxaparin with decreased risk (hazard ratio, 0.06; 95% CI, 0.010.74; p=0.03). The other variables were not associated with VTE.

The composite outcome of clinically suspected and confirmed VTE or hospital mortality occurred in 175/310 (56.5%) patients. The Cox regression analysis found age (hazard ratio per year increment, 1.027; 95% CI, 1.011-1.044), diabetes (hazard ratio, 1.652; 95% CI, 1.007-2.717) and the anticoagulant dose (intermediate-dose enoxaparin versus standard-dose UFH or enoxaparin hazard ratio, 0.421; 95% CI, 0.228-0.777; p=0.006) to be associated with the composite outcome of clinically suspected and confirmed VTE or hospital mortality. Therapeutic anticoagulation versus standard-dose UFH or enoxaparin was not associated with the composite outcome (hazard ratio, 1.008; 95% CI, 0.569-1.786). Similar results were noted when patients with suspected but not confirmed VTE were added to the confirmed VTE cases.

### Other outcomes

The clinical outcomes of patients with VTE compared with those who were not diagnosed to have VTE are shown in **Table 3**. There was no difference in major bleeding between UFH 5000 U 12 hourly versus 8 hourly (18.9% versus 17.0%, p=0.80) and between the standard-dose (10.6%) and intermediate-dose (8.8%) enoxaparin (p=0.71). Patients with VTE received more tracheostomy, had lower hospital mortality, and stayed longer in the hospital. The hospital mortality was lower even when patients with suspected but not confirmed VTE were added to the confirmed VTE cases (31.5% versus 50.8%, p=0.01). Patients who had arterial thrombotic events had similar hospital mortality compared with those who did not have these events (11/19 [57.9%] versus 136/291 [46.7%] patients, p=0.35).

**Table 3:** Outcomes of patients in the cohort

Variables	All patients N=310	VTE N=41	No VTE N=269	P value
Major bleeding, N (%)	42 (13.5)	8 (19.5)	34 (12.6)	0.23
Tracheostomy, N (%)	29 (9.4)	9 (22.0)	20 (7.4)	0.003
Duration of invasive MV (days), median (Q1, Q3)	12.0 (7.0, 20.0)	12.00 (7.0, 30.5)	12.0 (7.0, 19.0)	0.36
ICU LOS (days), median (Q1, Q3)				
All patients	10.0 (5.0, 18.0)	13.0 (7.0, 25.5)	10.0 (4.5, 17.0)	0.04
Patients who received invasive MV	14.0 (9.0, 22.0)	16.0 (9.5, 33.0)	14.0 (9.0, 21.0)	0.39
Hospital LOS (days), median (Q1, Q3)	19.0 (12.0, 29.0)	30.0 (19.0, 42.0)	17.0 (12.0, 27.0)	<0.001
ICU Mortality, N (%)	123 (39.8)	12 (29.3)	111 (41.4)	0.14
Hospital mortality, N (%)	147 (47.4)	13 (31.7)	134 (49.8)	0.03

ICU: intensive care unit; LOS: length of stay; MV: mechanical ventilation; Q1: first quartile; Q3: third quartile; RRT: renal replacement therapy; VTE: venous thromboembolism; Variables with skewed distribution were presented as median with the first third quartiles. The Mann-Whitney; U test was used to compare the two groups.

## Discussion

The main findings of this study were the following: anticoagulant thromboprophylaxis was provided in 97% of patients mostly as low-molecular weight heparin (LMWH); clinically suspected and confirmed VTE cases were common at 13.2% while arterial thrombotic events were less common at 6.1%; vasopressor therapy was associated with increased risk for VTE while intermediate-dose of enoxaparin versus standard-dose UFH or enoxaparin with decreased risk of the composite outcome of VTE or hospital mortality.

Thrombosis is highly prevalent in COVID-19. However, studies in the ICU setting showed variable results. For instance, one study of 184 ICU patients with COVID-19 pneumonia in three Dutch hospitals found that the cumulative incidence of a composite outcome of symptomatic acute PE, DVT, ischemic stroke, myocardial infarction or systemic arterial embolism was 49% (95% CI, 41-57%) [5]. On the other hand, VTE rates were much lower in a study from Milan (8.3% of 48 patients in the ICU) [7]. In the randomized controlled trial that compared intermediate-dose (N=276) with standard-dose enoxaparin (N=286) in ICU patients with COVID-19, symptomatic VTE occurred in 3.3% and 3.5% of patients, respectively [23]. In a meta-analysis, the VTE incidence was higher among patients in the ICU (27.9% versus 7.1% in the ward) [10]. We found that symptomatic VTE was diagnosed in 13.2% and arterial thrombosis in 6.1%. Other symptomatic VTE may have been overlooked as patients may have been too sick to have diagnostic workup or they were empirically treated with therapeutic anticoagulation. One meta-analysis showed that VTE incidence was higher when assessed according to VTE screening was performed (33.1% versus 9.8% by clinical diagnosis) [10].

Predictors of thrombosis in COVID-19 include age, prolonged prothrombin time > 3 seconds or PTT > 5 seconds [5], high D-dimerC [9] and central lines [6, 7]. We found that vasopressor therapy was associated with increased VTE risk on multivariable regression analysis. Admission D-dimer and D-dimer/fibrinogen ratio had only fair ability to discriminate VTE from non-VTE cases (area under the ROC curve of approximately 0.7 for both). One study found that D-dimer > 2,600 ng/mL predicted VTE (area under the ROC curve, 0.760; 95% CI, 0.661-0.858) with a sensitivity of 89.7%, and specificity of 59.5%.(9) In a meta-analysis, the pooled sensitivity of D-dimer for VTE in COVID-19 was 90% (95% CI, 90-90%) with a specificity of 60% (95% CI, 60-60%).

The procoagulant state in COVID-19 has been associated with worse outcomes. In 183 consecutive patients with severe COVID-19 pneumonia in China, the mortality rate was 11.5% with 71.4% of non-survivors having overt-DIC (≥ 5 points on the International Society on Thrombosis and Hemostasis diagnostic criteria) compared with 0.6% of survivors [31]. Hence, anticoagulants have been recommended in the management of COVID-19 to mitigate the micro- and macro-thrombotic complications before organ failure occurs [32], and to improve outcomes including survival. However, the counter argument is that in acute thrombotic microangiopathy, which results from hyperinflammation and endothelial damage as occurs in COVID-19 [3], treatment should be directed against the underlying disease and anticoagulation has a limited role [32, 34]. Multiple observational studies evaluated anticoagulants in COVID-19 with mixed results [5, 15, 35]. A recently published randomized controlled trial compared intermediate-dose and standard-dose enoxaparin in COVID-19 patients requiring ICU admission and found no difference in the primary efficacy outcome (a composite of venous or arterial thrombosis, treatment with extracorporeal membrane oxygenation, or mortality within 30 days of enrolment) between the two groups [22]. A collaboration between 3 independent international trial platforms (ATTACC, REMAP-CAP, and ACTIV-4) evaluating the safety and efficacy of therapeutic-dose versus standard dose thromboprophylaxis in hospitalized patients with COVID-19 recently reported their results [21]. Among patients requiring ICU admission for organ support, such as invasive medical ventilation or vasopressors, enrolment was halted for futility in December 2020 by the data and safety monitoring boards after 1123 patients had been enrolled [21]. In a multicenter randomized controlled trial, therapeuticdose LMWH reduced the composite outcome of VTE, arterial thromboembolism or death (relative risk, 0.68; 95% CI, 0.49-0.96; p=0.03) compared with standard heparin thromboprophylaxis among inpatients with COVID-19 and elevated D-dimer levels (> 4 fold the upper limit of normal) [21]. However, this treatment effect was not seen in ICU patients [20]. In the current study, different doses of anticoagulants were used; almost one third of patients received intermediate-dose enoxaparin; and patients with VTE had lower mortality. One explanation is that sicker patients or those who had Do-Not-Resuscitate orders, who typically have worse outcomes, did not have workup for VTE. In the multivariable regression analysis, intermediatedose LMWH was associated with lower risk or VTE or hospital mortality compared with standard-dose UFH or LMWH. Heparin use may have contributed to improved outcomes, through its anticoagulation effect or through other anti-inflammatory or antiviral mechanisms. In critically ill patients, standard-dose (40 mg) enoxaparin may yield subtherapeutic levels of anti-factor Xa [36], likely secondary to impaired absorption from vasopressor-mediated vasoconstriction, subcutaneous edema, and obesity. Hence, higher anticoagulant doses may be needed to have effective antithrombotic activity [36]. A recent meta-analysis of 24 trials found that anticoagulants had no significant effect on mortality in sepsis in general, but with significant reduction in mortality (risk ratio, 0.72; 95% CI, 0.62-0.85) in the subgroup of patients with sepsis‐induced DIC [37].

We did not observe increased risk of major bleeding with intermediate-dose LMWH versus prophylactic-dose heparin. Data about bleeding with anticoagulation in COVID-19 have shown different results. Prophylactic anticoagulation was not associated with increased risk of bleeding that required transfusion (hazard ratio, 0.87; 95% CI, 0.71-1.05) in one study [16]. Intermediate-dose enoxaparin did lead to higher rate of major bleeding in a trial [22]. A retrospective study which evaluated 105 patients hospitalized for COVID-19 pneumonia (63.8% severe) treated with various doses of subcutaneous enoxaparin (40-100 mg/ day) found uncommon hemorrhagic incidents (0% fatal hemorrhage and 1.9% major bleeding); and only one thrombotic event (PE) [38]. When compared to younger patients, patients older than 85 years did not have an increased risk of bleeding or need for blood transfusion [38]. However, a meta-analysis found an incidence of 7.8% (95% CI, 2.6-15.3) for bleeding, and 3.9% (95% CI, 1.2-7.9) for major bleeding [10]. The highest pooled incidence estimate of bleeding was reported for patients receiving intermediate- or full-dose anticoagulation (21.4%) [10]. The occurrence of major bleeding has been associated with high mortality [19].

The study findings should be interpreted considering its strengths and limitations. All patients in the current study were critically ill and our study reflected the practices of anticoagulant use early in the COVID-19 pandemic when there was paucity of good quality evidence with inconsistent recommendations and guidelines. We evaluated the composite outcome of VTE or hospital mortality to address ascertainment bias. The limitations include the retrospective analysis from a single center and the use of only admission variables. We also did not collect data about mechanical thromboprophylaxis. However, only 9 patients did not receive any form of heparin and adjunctive use of intermittent pneumatic compression has not been shown to reduce VTE risk [39]. Additionally, our analysis focused on cases with venous, but not arterial thrombosis. Confounders may have led to the observed associations as discussed earlier.

## Conclusions

In conclusion, we found that anticoagulant thromboprophylaxis was used for most critically ill patients with COVID-19 using different heparin doses. Clinically suspected and confirmed VTE was common occurring in 13.2% of patients, but arterial thrombosis was less frequent and was diagnosed in 6.1%. Intermediate-dose of enoxaparin versus standard-dose UFH or enoxaparin was associated with decreased risk of VTE and of the composite outcome of VTE or hospital mortality in patients with severe COVID-19.

## Abbreviations

CI: confidence interval
COVID-19: Coronavirus Disease 2019
DIC: disseminated intravascular coagulation
DVT: deep vein thrombosis
ICU: intensive care unit
LMOWH: low-molecular weight heparin
PE: pulmonary embolism
PTT: partial thromboplastin time
ROC: receiver operating characteristic
SOFA: Sequential Organ Failure Assessment
UFH: unfractionated heparin
VTE: venous thromboembolism
